# Diabetes management using a clinical guideline versus usual practice: a feasibility trial-based qualitative study among Ayurvedic practitioners and patients in Nepal

**DOI:** 10.1177/20420188261417088

**Published:** 2026-02-13

**Authors:** Kaushik Chattopadhyay, Nisha Rana, Shristi Karki, Prerok Regmi, Pradip Gyanwali, Vasudev Upadhyay, Bihungum Bista, Nikhil Tandon, Sanjay Kinra, Tuhin Kanti Biswas, Jo Leonardi-Bee, Michael Heinrich, Jeemon Panniyammakal, Sarah Anne Lewis, Sheila Margaret Greenfield, Meghnath Dhimal

**Affiliations:** Lifespan and Population Health, School of Medicine, University of Nottingham, Nottingham NG5 1PB, UK; The Nottingham Centre for Evidence-Based Healthcare: A JBI Centre of Excellence, Nottingham, UK; Nepal Health Research Council, Kathmandu, Nepal; Nepal Health Research Council, Kathmandu, Nepal; Nepal Health Research Council, Kathmandu, Nepal; Nepal Health Research Council, Kathmandu, Nepal; Department of Ayurveda and Alternative Medicine, Ministry of Health and Population, Kathmandu, Nepal; Nepal Health Research Council, Kathmandu, Nepal; Department of Endocrinology, Metabolism and Diabetes, All India Institute of Medical Sciences, New Delhi, India; Department of Non-Communicable Disease Epidemiology, London School of Hygiene and Tropical Medicine, London, UK; Department of Kayachikitsa, J. B. Roy State Ayurvedic Medical College and Hospital, Kolkata, West Bengal, India; Lifespan and Population Health, School of Medicine, University of Nottingham, Nottingham, UK; The Nottingham Centre for Evidence-Based Healthcare: A JBI Centre of Excellence, Nottingham, UK; Centre for Pharmacognosy and Phytotherapy, School of Pharmacy, University College London, London, UK; Chinese Medicine Research Center/Department of Chinese Pharmaceutical Sciences and Chinese Medicine Resources, College of Chinese Medicine, China Medical University, Taichung, Taiwan; Sree Chitra Tirunal Institute for Medical Sciences and Technology, Thiruvananthapuram, Kerala, India; Lifespan and Population Health, School of Medicine, University of Nottingham, Nottingham, UK; Institute of Applied Health Research, University of Birmingham, Birmingham, UK; Nepal Health Research Council, Kathmandu, Nepal

**Keywords:** Ayurveda, clinical practice guideline, evidence-based, management, Nepal, qualitative research, type 2 diabetes mellitus

## Abstract

**Background:**

Type 2 diabetes mellitus (T2DM) is common in Nepal, where many people seek primary care through Ayurveda, a widely practiced traditional medical system. However, concerns remain about variability in clinical practice and the quality of care provided by Ayurvedic practitioners. No evidence-based clinical practice guideline (EB-CPG) currently exists for managing T2DM in Ayurveda. To address this, an EB-CPG was developed, and a feasibility trial was conducted to inform a future definitive cluster randomized controlled trial evaluating whether the EB-CPG would improve T2DM management compared to usual clinical practice.

**Objectives:**

To explore the experiences and perspectives of Ayurvedic practitioners and patients regarding the two treatment groups (EB-CPG-based care versus usual clinical practice) and participation in the feasibility trial.

**Design:**

Constructivist qualitative study.

**Methods:**

Semistructured interviews were conducted with Ayurvedic practitioners and patients. Data were analyzed thematically using an inductive approach and the framework method.

**Results:**

A total of 41 Ayurvedic practitioners and patients were interviewed. Common themes identified across both groups included facilitators and challenges in the trial patient recruitment, challenges in usual clinical care, experiences with EB-CPG-based care (predominantly positive), ease and difficulties of monthly follow-ups, and pros and cons of patient diaries for assessing medication compliance. Practitioner-specific themes included the diverse populations they serve; patients’ motivations for choosing Ayurveda over Western medicine; their motivations for trial participation; balancing clinical duties with the trial demands; confusion about trial roles and responsibilities; and other trial-related issues such as understanding processes and documentation, support and communication, coordination with data collectors, and finances and other resources.

**Conclusion:**

The EB-CPG-based approach for managing T2DM by Ayurvedic practitioners in Nepal was well-received and perceived as more beneficial than usual clinical practice. Trial experiences and perspectives were largely positive; however, several challenges related to trial conduct should be addressed in future studies, including the definitive cluster trial.

## Plain language summary

Managing diabetes with or without a clinical guideline: views of Ayurvedic doctors and patients from a feasibility trial in Nepal

Type 2 diabetes is a common health problem in Nepal. Many people seek treatment through Ayurveda, a traditional system of medicine. However, there is no guideline to help Ayurvedic doctors manage diabetes, and the care provided can vary widely. To help improve care, researchers developed a clinical guideline based on the best available scientific evidence. They then ran a feasibility trial to help plan a larger study that will test whether the guideline improves diabetes management compared to usual practice. This study explored what Ayurvedic doctors and patients thought about the two types of care used in the feasibility trial: one based on the new guideline and the other following usual practice. Researchers also wanted to understand their experiences of taking part in the trial. Researchers interviewed 41 participants, including both doctors and patients. Key findings included what helped or made it difficult to recruit patients into the feasibility trial, challenges with usual care, mostly positive views about care based on the new guideline, ease and difficulties of monthly follow-up visits, and the pros and cons of using patient diaries to record medicine use. Doctors also talked about the wide range of patients they treat, why people choose Ayurveda over Western medicine, and their own reasons for joining the trial. They discussed challenges such as balancing regular duties with the trial work, confusion about their roles, understanding procedures and paperwork, communication and coordination, and access to support and resources. In conclusion, the new guideline-based approach for managing diabetes by Ayurvedic doctors in Nepal was well-received and seen as more helpful than usual care. Most experiences of the feasibility trial were positive, but some challenges in how the trial was carried out were identified. These should be addressed in future studies, including the planned larger trial.

## Introduction

Type 2 diabetes mellitus (T2DM) is a prevalent chronic condition that imposes a significant health and socioeconomic burden worldwide.^1,2^ Chronic hyperglycemia in this complex metabolic disorder is associated with a range of complications, including both macrovascular and microvascular damage, reduced health-related quality of life, and even premature death.^1,2^ In Nepal, the prevalence of T2DM increased from approximately 8% during 2010–2015 to 11% during 2015–2020.^3^ This figure is likely an underestimate, as many individuals remain undiagnosed due to the often asymptomatic nature of T2DM in its early stages.^4^ The rising burden of T2DM in Nepal contributes to increased morbidity and mortality and places significant strain on the health system, including higher service demand, escalating costs, and already strained infrastructure and limited workforce capacity. Weak primary care further exacerbates these challenges, leading to delayed diagnosis, increased risk of complications, and greater reliance on secondary and tertiary care services.^5,6^

Ayurveda, the dominant traditional medical system in Nepal, has been used for thousands of years for primary healthcare needs.^7^ Today, qualified and registered Ayurvedic practitioners are integral to public and private healthcare systems, often serving as primary clinical providers in Ayurveda centers.^7,8^ The basic principle of T2DM treatment is the same in Western and Ayurvedic medical systems: a combination of a healthy lifestyle and medicinal products.^9^ T2DM is one of the most common conditions for which individuals seek Ayurvedic care, and many people use Ayurvedic medicines containing ingredients of plant, animal, or mineral origin, either singly or in combination, consistently from the time of diagnosis.^8,10^ The main reasons for this include cultural health beliefs and a preference for avoiding side effects, costs, and administration modes associated with Western medicine (such as insulin injections), particularly among rural, impoverished, older, and Indigenous populations, and limited availability of Western medicine clinicians in rural areas.^10–15^

Although systematic reviews of clinical trials have shown the effectiveness and safety of Ayurvedic medicines in improving glycemic control and other vital outcomes in T2DM,^16,17^ concerns persist regarding suboptimal disease management by Ayurvedic practitioners in real-world settings.^13,18–23^ These concerns arise from the use of untested, ineffective, potentially unsafe, nonstandardized, or low-quality Ayurvedic formulations, which may lead to serious adverse effects, including heavy metal poisoning.^19,23–27^ Additionally, many practitioners follow unverified claims or adopt a trial-and-error approach to treatment.^22,28^ There are also instances where contradictory lifestyle advice is given, such as recommendations for ghee consumption (clarified butter made from saturated fat).^29^ Crucial steps in the care pathway, such as detection and management of poorly controlled T2DM and complications, including specialist referrals, are left to individual discretion, leading to unacceptable variations in clinical practice.^13,15,18,19,21–23^

Evidence-based clinical practice guidelines (EB-CPGs) are statements based on the best available scientific evidence, providing recommendations to support clinicians in decision-making and delivering quality care through effective and safe interventions.^30,31^ In essence, EB-CPGs serve as tools to translate scientific research into clinical practice.^30,31^ While CPGs in Western medicine have been shown to improve T2DM management,^32,33^ no such CPG exists for Ayurvedic practitioners in Nepal. Therefore, an EB-CPG may address existing problems, as its unavailability is a major challenge identified by several stakeholders, including these practitioners.^13,22,34–36^ To address this gap, we developed an EB-CPG and conducted a feasibility trial with the aim of informing a future definitive cluster randomized controlled trial to assess whether the use of the EB-CPG leads to better management of T2DM compared to usual clinical practice (i.e., without a CPG).^37,38^ The feasibility trial findings will be published elsewhere.^39^ The aim of this study was to explore the experiences and perspectives of Ayurvedic practitioners and patients regarding the two treatment groups (EB-CPG-based care versus usual clinical practice) and participation in the feasibility trial. The findings will inform refinements to the intervention and the design of a future definitive trial.

## Methods

This study employed a constructivist qualitative methodology, prioritizing the understanding of participants’ subjective experiences and perspectives within the healthcare context. Specifically, this approach enabled an in-depth exploration of how Ayurvedic practitioners and patients experienced and perceived the two treatment groups and the feasibility trial.

This qualitative study, conducted as part of the feasibility trial, was coordinated locally by the Kathmandu-based Nepal Health Research Council (NHRC). The trial was conducted in public and private Ayurveda centers (clusters), within and outside Kathmandu Valley. Each center had at least one Ayurvedic practitioner with a minimum of a 5½-year undergraduate medical degree in Ayurveda, registered with the Nepal Ayurveda Medical Council. People from diverse socioeconomic backgrounds visit these centers when they experience disease symptoms, and T2DM is often an incidental finding. When new, treatment-naïve T2DM cases were detected by participating practitioners, these adults were invited to participate in the trial.

In the intervention group, Ayurvedic practitioners managed T2DM using the EB-CPG developed by our team, available in both hard and soft copies.^37^ Briefly, we developed a high-quality, comprehensive EB-CPG for managing T2DM using a robust methodology that incorporated the best available scientific evidence and involved key stakeholders, including practitioners. EB-CPG covers T2DM diagnosis and treatment targets, lifestyle advice, Ayurvedic antidiabetic medicines, complications screening and management, and specialist referrals for poorly controlled T2DM and its complications. To support its uptake and adherence, practitioners received (i) regular face-to-face and online group training in using the EB-CPG, including individual role-playing and feedback and peer-led sessions to consolidate learning, and (ii) an EB-CPG-based structured booklet for writing case notes. Additionally, an EB-CPG-recommended, standardized, quality-controlled, plant-based Ayurvedic antidiabetic medicine (capsule) was made available at the centers and provided free of charge to patients for the duration of the study. In the control group, usual clinical practice continued, with practitioners managing T2DM without using a CPG.

Semistructured qualitative interviews were conducted with Ayurvedic practitioners and patients in 2023–2024. Intervention and control group practitioners at all trial sites were approached at the end of their participation, either face-to-face or by telephone, by local female multilingual health researchers trained in qualitative research (N.R., Sh.K., or A.A.). Similarly, patients were approached at the end of their trial participation using purposive sampling to ensure diversity in the treatment group (intervention or control), age, gender, and geographical location (inside or outside Kathmandu Valley). The sample size was determined based on recommendations to allow for data saturation.^40^ To improve the range of perspectives in sampling, we also approached those who declined to participate in the trial or who were recruited but later withdrew. Researchers provided a participant information sheet and a verbal study description and answered any questions. Written informed consent was obtained from those who expressed interest. Participant information sheets and consent forms were available in Nepali and English.

With prior appointments, one-on-one interviews were conducted separately with Ayurvedic practitioners and patients, with data collection from both groups occurring in parallel. Most interviews were conducted by N.R., with some by Sh.K. and A.A. N.R. and A.A. were independent of the trial. Sh.K. was the trial coordinator, which may have influenced data collection. However, her detailed knowledge of the trial was beneficial during interviews, allowing for more specific probing and discussion with participants. The timing and location of interviews (i.e., face-to-face at the center or home or via telephone) were arranged according to the interviewees’ convenience. Interviews were conducted in Nepali, the participants’ preferred language. Predeveloped and pretested interview topic guides were used, and interviews were audio recorded. Field notes were also taken to capture nonverbal cues such as gestures and tone of voice, which might not have been adequately captured through audio recording.

Based on the study’s aim and existing relevant literature, three interview topic guides were developed: one each for completing and noncompleting Ayurvedic practitioners, completing and noncompleting patients, and those who declined trial participation. These guides were refined by the multidisciplinary research team. They covered topics such as provision and receipt of usual clinical care and EB-CPG-based care for managing T2DM, depending on the treatment group, as well as recruitment and participation in the trial. The topic guide for those who declined participation was shorter and focused mainly on exploring their reasons for nonparticipation if they agreed to be interviewed. Guides allowed interviewees to freely express their views, with follow-up questions and probes to capture further detail and develop a deeper understanding of their responses. Topic guides were translated into Nepali by a professional translator based at NHRC and pretested among a small sample of external practitioners and patients to ensure the appropriateness of meaning and understanding.

Professionals at NHRC transcribed the interview recordings verbatim and translated them into English. The qualitative researcher (N.R.) listened to interview recordings multiple times, corrected any inaccuracies in the final transcripts, and anonymized them by redacting identifiable information. Anonymized transcripts were imported into NVivo 14 software for data storage and organization.

Data were analyzed thematically using an inductive approach and the framework method to align with the study’s aim and capture emergent issues raised by the interviewees.^41,42^ Separate but concurrent analyses were performed for Ayurvedic practitioners and patients. Two researchers (K.C. and N.R.) independently familiarized themselves with the interview transcripts through multiple readings and performed line-by-line coding. Preliminary codes were discussed collaboratively, and discrepancies were resolved through consensus rather than formal intercoder reliability statistics, consistent with qualitative best practices. Codes were grouped into categories and then organized into subthemes and overarching themes, refined iteratively as new data emerged, until data saturation was reached—that is, no new themes were identified. Field notes provided additional context and depth to the interpretation of interview data.^43^ Final themes for both groups were compared to identify similarities and differences.

The analysis was led by K.C., who has extensive experience in qualitative research, is the principal investigator of the trial, and is a trained Ayurvedic practitioner from the neighboring country, India. He is familiar with the context in Nepal, including population characteristics, health-related beliefs and cultural practices, and the structure of the health system. K.C. worked alongside N.R., an independent qualitative researcher based in Nepal, and in consultation with S.M.G., a senior qualitative researcher and methodologist with a background in medical sociology. While K.C.’s dual role may have influenced interpretation, it also ensured that the analysis remained closely aligned with the Nepalese context and the practical realities of Ayurvedic care. Nonattributable verbatim quotes are used to illustrate the core ideas of each theme and subtheme.

This study is reported in accordance with the Consolidated Criteria for Reporting Qualitative Research (COREQ).^44^

## Results

We interviewed 41 individuals (see Table 1): 7 Ayurvedic practitioners from the intervention group and 7 from the control group, 10 patients from the intervention group and 10 from the control group, and a few individuals who declined to participate in the trial or were recruited but later withdrew (1 practitioner and 6 patients). Among the practitioners, six were female, and nine were male. The patients had an average age of 51 years (ranging from 38 to 69 years), with an equal distribution of females and males (13 each) and those residing inside and outside Kathmandu Valley (13 each). The interviews lasted between 20 and 80 min, with an average duration of 45 min.

**Table 1.** Individuals interviewed.

	Recruited and completed the trial	Declined to participate in the trial	Recruited in the trial but later withdrew
Ayurvedic practitioners in the intervention group	7	n/a	n/a
Ayurvedic practitioners in the control group	7	n/a	1^a^
Patients in the intervention group	10	1	2
Patients in the control group	10		3

a Ayurveda center didn’t recruit patients and left the study.

n/a, not applicable.

See Table 2 for Ayurvedic practitioner and patient themes and subthemes (quotes are in Table 3).

**Table 2.** Participant themes and subthemes.

Ayurvedic practitioners	Patients
Facilitators for patient recruitment in the trial	
• Trust and reputation of the Ayurveda centers • Free tests and medicines as incentives • Positive perception of Ayurvedic medicines and the trial’s assurance of safety • Word-of-mouth referrals and growing demand from enrolled patients	• Trust in Ayurvedic medicines • Social influence and recommendations • Perceived benefits of the trial • Clarity about the trial and related processes
Challenges in patient recruitment in the trial	
• Patients’ hesitancy and mistrust of research • Gender-based differences in decision-making • Concerns about continuity of clinical care posttrial • Poor comprehension of the trial consent process • Value of regular recruitment strategy discussions • Need for adjusting the trial eligibility criteria	Poor comprehension of the trial consent process
Challenges in providing and receiving usual clinical care	
• Variability and concerns in clinical practice and the care pathway • Lack of clear lifestyle advice • Issues with Ayurvedic medicines’ effectiveness, forms, quality, and availability	• Confusion and uncertainty about lifestyle changes • Issues with Ayurvedic medicines’ effectiveness, forms, quality, and availability • Confusion and lack of clarity on Ayurvedic medicines
Experiences of providing and receiving EB-CPG-based care, which were predominantly positive	
• EB-CPG training and support • Adherence to the EB-CPG and its usefulness • EB-CPG-based structured booklet for writing case notes • Demand for a patient booklet to complement EB-CPG-based lifestyle advice • Demand for continued EB-CPG-recommended Ayurvedic antidiabetic medicine	• Positive interactions with Ayurvedic practitioners • Effective communication and clear instructions • Adherence to EB-CPG-based lifestyle advice • Compliance with EB-CPG-recommended Ayurvedic antidiabetic medicine • Routine medication adherence challenges • Perceived benefits and increased trust in Ayurveda • Supply of EB-CPG-recommended Ayurvedic antidiabetic medicine • Demand for a patient booklet to complement EB-CPG-based lifestyle advice • Demand for continued EB-CPG-recommended Ayurvedic antidiabetic medicine
Ease and difficulties of monthly follow-ups	
• Improving follow-up adherence • Challenges with timing and scheduling	• Ease of monthly visits • Challenges with timing and health issues • Poor comprehension of the processes
Pros and cons of maintaining patient diaries for assessing compliance with EB-CPG-recommended Ayurvedic antidiabetic medicine	
• Utility and challenges in daily use • Literacy and understanding issues • Data accuracy	• Ease of use and convenience • Challenges faced by busy or illiterate patients • Forgetfulness and stress in diary maintenance • Diary-related logistics
Ayurvedic practitioners serve diverse populations	
• Socioeconomic diversity • Age range of diabetes patients	
Patients’ motivations for choosing Ayurveda over Western medicine	
Ayurvedic practitioners’ motivations for participating in the trial	
Balancing clinical duties with the trial demands	
Confusion regarding roles and responsibilities in the trial	
Issues in understanding the trial processes and documentation	
Issues in the trial support and communication	
Issues in coordination with the trial data collectors/outcome assessors	
Trial-related financial and other resource issues	

EB-CPG, evidence-based clinical practice guideline.

**Table 3.** Participant quotes.

Ayurvedic practitioners and patients	
Facilitators for patient recruitment in the trial	
Trust and reputation of the Ayurveda centers	• “Recruitment was easier due to our hospital’s 14-year reputation and patient trust.” (MAP-CG) • “High patient flow at our center made recruitment easier.” (MAP-IG)
Free tests and medicines as incentives	• “Offering free tests and medicines helped convince patients.” (MAP-CG)
Positive perception of Ayurvedic medicines and the trial’s assurance of safety	• “The belief that Ayurvedic medicine has no side effects helped persuade patients to participate.” (FAP-IG) • “We didn’t have to prescribe untested medicine, which reassured patients.” (MAP-IG)
Word-of-mouth referrals and growing demand from enrolled patients	• “Some enrolled patients requested enrollment of other new diabetes patients.” (MAP-IG)
Trust in Ayurvedic medicines	• “I trust Ayurvedic medicines. Since I was familiar with the doctor, I specifically asked him to prescribe only Ayurvedic medicines, not allopathic ones.” (MP-CG) • “I’ve taken Ayurvedic medicine before, even for high blood pressure. I’ve preferred it since childhood and now give it to my kids.” (FP-IG) • “I realized Ayurvedic medicine can work quickly, so I promised myself to avoid allopathic medicine.” (MP-CG)
Social influence and recommendations	• “My friends say allopathic treatment is harmful. First, you get sugar, and later, cholesterol. They advised that Ayurvedic treatment is better—more sustainable and cheaper than allopathy.” (MP-IG) • “A neighbor’s high sugar level didn’t respond to allopathy, but Ayurvedic medicine helped bring it under control.” (FP-CG) • “When someone is newly diagnosed with diabetes, I recommend Ayurvedic medicine.” (FP-CG) • “I’ve heard that once you start allopathic medicine, you have to take it continuously and can’t stop.” (MP-CG)
Perceived benefits of the trial	• “The world has so many people with diabetes. If this kind of research continues and comes to Nepal, it will be very beneficial, especially since it’s being done free of cost now.” (MP-IG) • “This new, safe treatment technique is being tested on me, and I believe many others will benefit too.” (MP-IG)
Clarity about the trial and related processes	• “I understood the study well. If there was anything I didn’t understand, I asked him, and he explained it to me.” (MP-IG) • “It wasn’t that difficult. My husband and I came together, and he understood everything.” (FP-CG)
Challenges in patient recruitment in the trial	
Patients’ hesitancy and mistrust of research	• “Many patients were apprehensive about ‘research’ despite already using my Ayurvedic prescriptions.” (MAP-CG) • “A patient asked if he was being made a ‘guinea pig’ and wanted assurance that his health wouldn’t suffer.” (MAP-IG) • “One patient was anxious, fearing we might take his property, even after a clear consent explanation.” (FAP-CG) • “I struggled to enroll some older patients with limited understanding, even when they met criteria.” (MAP-IG)
Gender-based differences in decision-making	• “Male patients decided quickly, but female patients often needed family permission.” (FAP-CG)
Concerns about continuity of clinical care post-trial	• “They were concerned about whether the medicine would be available outside the trial.” (FAP-IG)
Poor comprehension of the trial consent process	• “Most patients didn’t read the consent form; some needed it read to them, and one didn’t understand it at all.” (FAP-CG)
Value of regular recruitment strategy discussions	• “Regular meetings with Dr ABC (Principal Investigator) were helpful for updates. More frequent meetings, every 4–6 months or online, would have been beneficial to know about each other’s progress, challenges, and recruitment strategies.” (FAP-CG)
Need for adjusting the trial eligibility criteria	• “The HbA1c limit would be better set at 10 instead of 9, as Ayurveda can manage up to 10.” (MAP-CG)
Poor comprehension of the trial consent process	• “It was a bit difficult for me to understand since I am not very literate. She helped by repeating things I found confusing, and I eventually understood.” (MP-CG) • “I didn’t read the consent form because I already knew the doctor well and trusted him. He explained everything to me, and I signed the paper.” (MP-CG) • “I signed the consent form in Nepali, but I didn’t fully understand it, even after looking over it a few times.” (FP-CG)
Challenges in providing and receiving usual clinical care	
Variability and concerns in clinical practice and the care pathway	• “Fixed standard drugs would ensure uniformity; currently, one Ayurvedic doctor might prescribe differently than another.” (MAP-CG) • “Ayurvedic doctors prescribe a range of Ayurvedic medicines. The medicines used can be somewhat vague, and it is difficult to know their limitations and benefits. Clearer treatment protocols would simplify practice.” (MAP-IG) • “Ayurvedic drugs are effective when HbA1c is below 9. For higher levels, it’s better to start with allopathic drugs and switch to Ayurveda later. I’ve managed diabetes for top politicians in Nepal, transitioning them from insulin to Ayurvedic treatment.” (MAP-CG)
Lack of clear lifestyle advice	• “We haven’t been able to give them a clear guidance on what they should eat and avoid.” (MAP-CG)
Issues with Ayurvedic medicines’ effectiveness, forms, quality, and availability	• “If good medicines aren’t available and only weaker ones are, the patient won’t return after using it once.” (MAP-CG) • “We should provide extracted active ingredients instead of powdered herbs, as tablets are easier for patients to consume.” (MAP-CG) • “Medicine availability is an issue. Some patients accept alternatives, but others get angry. I must also acknowledge that quality of local medicines is low.” (FAP-CG) • “Our hospital distributes government-provided free medicines, but patients can also purchase them from our pharmacy.” (MAP-CG)
Confusion and uncertainty about lifestyle changes	• “I’m upset I can’t eat rice, and I feel too stressed to cook alternatives. If I can’t eat potatoes, it feels like there are no other vegetables available. What should I eat daily?” (FP-CG)
Issues with Ayurvedic medicines’ effectiveness, forms, quality, and availability	• “I’m receiving the medicine in powder form for free. I have to buy the tablets because the free tablets didn’t work.” (FP-CG) • “I took the capsules regularly for 4 months but then switched to the powder form when the capsules were unavailable. The powder is made from Indian gooseberry, but there are different types available. I don’t think the powder is as effective as the capsules. Sometimes, I miss taking the powder.” (MP-CG) • “I can’t tell if it’s the same medicine since the bottle and flavor have changed.” (FP-CG) • “I initially took the medicine from here but then had to buy it from another pharmacy because Himalayan Ayurvedic medicine was not available.” (FP-CG) • “The doctor gave my mother capsules and bitter liquids, but none of them helped reduce her sugar levels.” (MP-CG)
Confusion and lack of clarity on Ayurvedic medicines	• “There should be better follow-ups and clearer instructions on how to use the medicines. Every patient cannot understand if they give advice once, that is why the advice needs to be repeated twice or thrice. If we ask them twice or thrice, then the doctor becomes angry. I was told my blood sugar level is still not controlled, but no further guidance was given.” (FP-CG) • “I wasn’t clearly instructed whether to continue with the powder or buy more capsules.” (MP-CG)
Experiences of providing and receiving EB-CPG-based care, which were predominantly positive	
EB-CPG training and support	• “ABC sir (Principal Investigator) regularly trained and updated us with online and in-person sessions.” (MAP-IG) • “We received training on advising patients and handling complications, ensuring we were ready for their questions.” (MAP-IG) • “ABC sir (Principal Investigator) briefed us on the medication, its function, and the research, which gave us confidence and helped persuade patients.” (MAP-IG)
Adherence to the EB-CPG and its usefulness	• “Sticking to the guideline ensured consistency across our work.” (MAP-IG) • “I advise patients on medication, diet, exercise, and lifestyle modifications according to the guideline.” (MAP-IG) • “Previously, I didn’t routinely discuss diabetic foot and retinopathy unless patients had complications. Now, I automatically remember to advise on these issues. Overall, it has helped to manage patients better.” (MAP-IG) • “Following the guideline, I referred a patient because his HbA1c level increased.” (FAP-IG) • “Guideline-based management is more structured than usual care, which is often vague.” (FAP-IG) • “I like the concept of clinical guidelines. The guideline was well-planned, easy to follow, and helped refresh our knowledge.” (MAP-IG) • “The clear guideline minimized errors and deviations.” (FAP-IG) • “The guideline made it easier to explain things to patients and build trust.” (FAP-IG) • “Following the guideline benefits both patients and doctors with clear instructions. It improves patient health and encourages follow-ups, becoming part of our routine for managing type 2 diabetes.” (MAP-IG)
EB-CPG-based structured booklet for writing case notes	• “The prescription pad included essential information aligned with the clinical guideline, presented in the correct sequence.” (FAP-IG)
Demand for a patient booklet to complement EB-CPG-based lifestyle advice	• “I felt it would have been better if we’d provided a booklet on what patients should eat and do, along with our advice.” (MAP-IG)
Demand for continued EB-CPG-recommended Ayurvedic antidiabetic medicine	• “Patients progressed well and asked for more medicine post-trial, but I had to switch it, explaining it was only for research.” (MAP-IG) • “After 6 months, there’s no plan for continuity. How will patients trust us if we leave them after this?” (MAP-IG)
Positive interactions with Ayurvedic practitioners	• “He respects me and gives me time. The service here is very good. I’m getting better and have no plans to go elsewhere.” (MP-IG) • “They measured my weight, belly fat, blood sugar, and pressure every month.” (FP-IG)
Effective communication and clear instructions	• “The doctor explained the dos and don’ts in simple language.” (FP-IG) • “They told me to take the medicine at the same time every day and not miss any doses.” (FP-IG) • “He explained the blood reports, showing me the previous and current results to ensure I understood since I didn’t know how to read these.” (FP-IG)
Adherence to EB-CPG-based lifestyle advice	• “I lost weight in a month by avoiding oily food and starting exercise, including cycling.” (FP-IG) • “I’ve cut down fat, salt, and sugar, eat more fruit, and exercise regularly, strictly following their advice.” (MP-IG) • “I realized I should exercise regularly, whether in the morning or evening. I now consistently do yoga and exercise. I used to eat excessively, but now I eat less and focus on health rather than just taste.” (MP-IG)
Compliance with EB-CPG-recommended Ayurvedic antidiabetic medicine	• “I need to work, so I haven’t missed a single day of medication. If I have to go somewhere, I prepare a packet and take it with me.” (MP-IG) • “I’ve developed a habit of taking my medicine regularly. It’s important to have discipline, which I’ve now achieved.” (MP-IG)
Routine medication adherence challenges	• “Sometimes, when I’m in a hurry, I forget to take my medicine with me.” (MP-IG) • “I went to a program and thought I would return quickly, so I didn’t carry my medicine. I didn’t forget to take it; I just forgot to bring it with me.” (FP-IG) • “Occasionally, it becomes irritating when you have to take too many medications.” (FP-IG)
Perceived benefits and increased trust in Ayurveda	• “I’m very happy the medicine worked for my problems. When my sugar levels were high, we were worried, but this medicine brought them under control. I didn’t need any other medicine, and the capsules were easy to take.” (FP-IG) • “Other medicines have side effects, but this one didn’t.” (MP-IG) • “The medicine, along with monthly advice and follow-ups, has improved my health. If this kind of Ayurvedic treatment could be expanded to other diseases, it would be beneficial.” (MP-IG) • “This treatment has increased my trust in Ayurveda and made me more conscious about my health.” (MP-IG)
Supply of EB-CPG-recommended Ayurvedic antidiabetic medicine	• “They only provided medicines for up to a month at a time, and sometimes I was late due to my schedule.” (FP-IG) • “One week before my medicine ran out, they called to check if I could come. When I said I couldn’t, they delivered the medicine to my home.” (FP-IG) • “If people receive too much medicine at once, they may not take it on time. A 1-month supply is adequate.” (FP-IG)
Demand for a patient booklet to complement EB-CPG-based lifestyle advice	• “I had seen this paper (EB-CPG), but it wasn’t provided to me. I took a photo of it to know what I should and shouldn’t eat.” (MP-IG)
Demand for continued EB-CPG-recommended Ayurvedic antidiabetic medicine	• “These 6 months of treatment won’t be enough for us. Will the research continue to support future treatments or blood tests? If not, all my participation might go to waste. This research should either continue fully or not at all.” (MP-IG) • “I liked everything, but now I’m wondering what to do once the medicine is finished.” (FP-IG) • “The treatment helped me, and I shared my experience with others, but by then the medicine was no longer available. There are other newly diagnosed diabetes patients, and I asked the doctor, but they said it was finished. If it were available everywhere, it would be more beneficial.” (FP-IG)
Ease and difficulties of monthly follow-ups	
Improving follow-up adherence	• “Regular calls or messages could help reduce dropouts.” (FAP-CG) • “Patients who see improvements and understand the long-term nature of the disease tend to continue follow-ups, even if not always on time.” (MAP-IG) • “Monthly blood test reports built trust, which would be hard to achieve without check-ins.” (FAP-IG) • “I prioritized them, so they didn’t have to wait for reports, which made them feel privileged and cared for.” (MAP-CG) • “We called for regular follow-ups and ensured patients received their medicine on time to avoid missed doses.” (MAP-IG)
Challenges with timing and scheduling	• “Requiring monthly follow-ups within a 5-day window was challenging. Patients from Rolpa travel a day to reach here, and some asked to come every 1.5–2 months instead.” (MAP-CG) • “Community fairs and festivals cause delays in testing and follow-ups, with some patients arriving late.” (FAP-CG) • “Coordinating monthly follow-ups with OPD and IPD duties was difficult.” (FAP-IG) • “It’s impractical for doctors to repeatedly call patients who miss follow-ups. I ask receptionists to check on them, but they’re also busy.” (FAP-CG)
Ease of monthly visits	• “There were no difficulties during the monthly visits. Initially, I felt a bit lazy, but later, I realized the importance of taking care of my health, and it became a habit. The center is nearby, so it wasn’t difficult for me. They scheduled my appointments during the daytime when there were fewer people, so I could easily get my check-up and save time.” (MP-CG) • “I took an auto for Rs. 100 each way, but it wasn’t difficult since it was for my health.” (FP-IG) • “Even if I asked to come the next day, they managed to see me the same day.” (FP-IG) • “She would call me when it was time for follow-ups and sometimes remind me in between. I had to adjust my schedule because of work, but she would still accommodate me, even on holidays.” (MP-IG)
Challenges with timing and health issues	• “Monthly visits are fine when I’m home, but it’s hard when I’m away and managing household duties.” (FP-IG) • “I had difficulty attending monthly follow-ups due to high blood pressure and gastric issues, especially since the clinic opens at 10 a.m. and I had to come on an empty stomach. Although they arranged transportation when I was unwell, it was still challenging.” (FP-IG)
Poor comprehension of the processes	• “I assumed it was fine to do the blood tests elsewhere. For 1 or 2 months, I did the test outside. It might be because I didn’t understand it properly.” (FP-CG)
Pros and cons of maintaining patient diaries for assessing compliance with EB-CPG-recommended Ayurvedic antidiabetic medicine	
Utility and challenges in daily use	• “The daily medicine booklet was useful. It helped to track whether patients took their medicine regularly, integrating well into their daily routine.” (MAP-IG) • “Patients didn’t maintain diaries despite taking their medicines; they often forgot to fill them in, finding the daily diary concept challenging.” (MAP-IG)
Literacy and understanding issues	• “Many patients struggled with the diary, especially those who were illiterate, viewing it as a burden, which may have led to drop-outs.” (MAP-IG)
Data accuracy	• “I sometimes filled in the diaries, asking patients to report their medicine intake, and provided new bottles only after receiving the empty ones for accuracy.” (MAP-IG) • “One patient lost the diary, and some lied about entries out of fear. We tracked adherence by counting medicines, and they admitted mistakes when questioned.” (FAP-IG)
Ease of use and convenience	• “I filled out the diary daily, keeping it with my medicine on my bedside shelf.” (FP-IG) • “It wasn’t too difficult because it was explained to me. I would make notes if I missed a day and update it later. The diary was useful for remembering doses; checking off each day helped keep track.” (FP-IG)
Challenges faced by busy or illiterate patients	• “I didn’t find it difficult, but it might be hard for other patients who are illiterate or very busy.” (FP-IG) • “I requested the doctor to fill in the diary for me.” (MP-IG)
Forgetfulness and stress in diary maintenance	• “Having a disease is stressful, and taking medicine and marking it can be troublesome. Sometimes, I forgot to mark it. Since I’m working, I occasionally ticked off for 5 days, a week, or a month altogether. As I took the medicine regularly, I remembered.” (MP-IG)
Diary-related logistics	• “The diary was quite small. A larger diary would have provided more space for entries over the 6 months.” (FP-IG) • “No, they didn’t give me a diary. I didn’t notice it and didn’t even know about it.” (FP-IG)
**Ayurvedic practitioners**	
Ayurvedic practitioners serve diverse populations	
Socioeconomic diversity	• “We see patients from various socioeconomic backgrounds, including lower middle to upper middle classes.” (MAP-IG) • “In government hospitals, we mostly see patients from poorer economic backgrounds.” (MAP-CG)
Age range of diabetes patients	• “We now see very young diabetes patients, as young as 25–26 with borderline diabetes, though most are still above 50.” (FAP-IG)
Patients’ motivations for choosing Ayurveda over Western medicine	
	• “Most patients believe Ayurvedic medicines have no side effects, even long-term, and encourage others to try it after successful treatment.” (FAP-IG) • “Patients with high blood glucose often ask to switch to Ayurvedic medicines, but I advise them to stabilize with allopathic treatment first.” (MAP-CG) • “When patients on metformin or other allopathic drugs hear they might need to start insulin, they become anxious and seek Ayurvedic alternatives.” (FAP-CG) • “Some insulin users seek Ayurvedic alternatives due to high costs.” (MAP-CG)
Ayurvedic practitioners’ motivations for participating in the trial	
	• “Evidence-based clinical practice is crucial. As Ayurveda becomes more evidence-based, it will benefit both the field and Ayurvedic doctors.” (MAP-IG) • “I trust what I see and experience. My interest in research grew during my MD studies due to its data-driven nature, and participating in an international diabetes study was an added motivator.” (MAP-CG) • “I was excited to contribute to Ayurveda research for noncommunicable diseases, as it can yield effective results.” (MAP-IG) • “Research is needed to improve disease management, which is lacking in our system. Ayurvedic practitioners need an open mindset and knowledge of different treatment options.” (MAP-CG) • “This trial is more about a treatment protocol than just the medicine itself.” (FAP-IG) • “This is the first time in Nepal’s history that research on Ayurveda is being conducted in this way.” (MAP-IG) • “Solid evidence would make it easier to convince patients about Ayurveda’s benefits, and good results will attract more people.” (FAP-IG)
Balancing clinical duties with the trial demands	
	• “Research isn’t part of our usual job. It added to our daily workload, especially with high patient volumes. Many saw it as extra work, so we managed by working longer or sacrificing leisure. Despite the challenges, we learned a lot.” (MAP-IG) • “At times, we had to pause regular work; additional manpower would have helped.” (FAP-IG)
Confusion regarding roles and responsibilities in the trial	
	• “Even though roles were defined, it was unclear who to contact for different issues. When I approached Dr ABC (a ‘blind’ data collector/outcome assessor) about certain matters, he told me it shouldn’t be discussed with him.” (FAP-CG) • “A doctor appointed for this job initially treated his responsibilities lightly, like homework, but we had to explain their importance.” (MAP-IG)
Issues in understanding the trial processes and documentation	
	• “NHRC hosted a program at the start, making it easier for us to understand. They also taught us how to explain it to patients, with instructions in both Nepali and English.” (MAP-CG) • “I found the research process complicated and had trouble helping patients understand it.” (FAP-IG) • “The orientation training wasn’t clear on protocols, and the paperwork was confusing. I figured things out as I went, but the training should have covered more practical scenarios.” (FAP-IG) • “I felt the paperwork was an additional burden, especially with the extensive documentation required.” (MAP-IG)
Issues in the trial support and communication	
	• “The support was good. If there was any confusion, I would call them, and they would answer and help us as much as possible.” (MAP-IG) • “She (Trial Coordinator) calls regularly and asks for updates, which I really liked.” (FAP-CG) • “Dr ABC (Trial Coordinator) visited frequently to check everything.” (FAP-CG) • “Coordination was excellent during recruitment, but communication decreased afterward. More frequent communication would have been helpful.” (FAP-IG) • “There were communication gaps. Some people with no experience in research were completely dependent on NHRC. More frequent follow-ups from NHRC would have helped.” (MAP-IG) • “Regular checks by NHRC would have been helpful, but they visited only once.” (FAP-IG)
Issues in coordination with the trial data collectors/outcome assessors	
	• “The outcome assessment team was considerate of patients’ availability, which was helpful.” (FAP-CG) • “The most challenging aspect was coordinating the timing of the outcome assessor due to their existing schedule.” (FAP-IG)
Trial-related financial and other resource issues	
	• “Dr ABC (Trial Coordinator) explained that the delay in payments was due to government protocols, but it would have been better if payments had been made on time.” (FAP-CG) • “NHRC requires bills for payment instead of advance payments, which often leads to out-of-pocket expenses and delays in reimbursement.” (MAP-IG) • “The prescription pad was delayed, and materials were provided later than expected.” (MAP-IG) • “The initial patient diary could only document information for one to two months, so I had to provide up to three diaries per person. Later, it was redesigned to cover 6 months.” (FAP-IG)

Green shading = Ayurvedic practitioners; Orange shading = patients.

CG, control group; EB-CPG, evidence-based clinical practice guideline; FAP, female Ayurvedic practitioner; FP, female patient; IG, intervention group; MAP, male Ayurvedic practitioner; MP, male patient.

### Themes common to both Ayurvedic practitioners and patients

#### Facilitators for patient recruitment in the trial

Ayurvedic practitioners pointed out that the reputation of the Ayurveda centers and high patient flow facilitated patient recruitment in the trial. Offering free tests and medicines encouraged enrollment, while a positive perception of Ayurvedic medicines and assurance of safety reassured patients. Additionally, word-of-mouth referrals from enrolled patients helped boost participation.

Patients reported that they were drawn to the trial by their trust in Ayurvedic medicines, social endorsements, and perceived benefits of free treatment. Many had positive personal experiences with Ayurveda and viewed it as safer and more sustainable than Western medicine. Social influences reinforced this view, and some patients saw their involvement as part of a valuable, potentially impactful initiative. Clarity of the trial and related processes also facilitated patient recruitment, with Ayurvedic practitioners playing a crucial role in helping them understand.

#### Challenges in patient recruitment in the trial

Ayurvedic practitioners highlighted recruitment challenges, including patient hesitancy and mistrust of research, concerns about continuity of clinical care posttrial, and poor comprehension of the consent process. Gender-based decision-making also influenced participation, with female patients often requiring family consent. Practitioners emphasized the need for regular discussions on recruitment strategies and suggested adjusting the trial eligibility criteria to better suit Ayurvedic treatment capabilities.

Patients, especially those with lower literacy levels, had difficulty understanding the consent process. They often signed the consent form based on their trust and familiarity with Ayurvedic practitioners.

#### Challenges in providing and receiving usual clinical care

Ayurvedic practitioners highlighted variability and concerns in clinical practice and the patient care pathway, noting the lack of clear lifestyle guidance for patients and limited knowledge of effective and safe Ayurvedic medicines to prescribe. Issues with medicine effectiveness, form, quality, and availability also led to patient dissatisfaction and impacted adherence.

Patients emphasized that they experienced inconsistencies in clinical care, including ambiguous guidance on lifestyle changes and medication use, which left them feeling confused and uncertain. They also highlighted issues with ineffective and poor-quality Ayurvedic formulations in varying forms, further complicated by availability challenges.

#### Experiences of providing and receiving EB-CPG-based care, which were predominantly positive

Ayurvedic practitioners noted that they received regular training through online and in-person sessions, which helped them confidently advise patients, prescribe EB-CPG-recommended Ayurvedic antidiabetic medicine, and handle complications. Practitioners highlighted that adherence to the EB-CPG minimized errors and ensured consistency in T2DM care, addressing lifestyle modifications, medication, complications, and specialist referrals. The EB-CPG was widely regarded for its structured, comprehensive, and straightforward approach, contrasting with the vagueness of usual clinical practice. It was well-planned, easy to follow, and helped refresh practitioners’ knowledge. EB-CPG made it easier to explain treatment and build trust with patients by improving patient care and enhancing communication. Several practitioners reported that EB-CPG became an integral part of their routine clinical practice posttrial, and they continued delivering EB-CPG-based care. Practitioners observed that the structured booklet for writing case notes provided a clear, sequenced format aligned with the EB-CPG, supporting standardized documentation in patient care. Some practitioners suggested a written booklet for patients to support EB-CPG-based lifestyle advice. Practitioners noted that several patients strongly desired to continue EB-CPG-recommended Ayurvedic antidiabetic medicine after the trial, but the lack of a continuity plan raised concerns about sustaining their trust.

Patients highlighted that they valued respectful interactions with Ayurvedic practitioners and clear guidance on lifestyle and medication. Regular health monitoring helped them feel supported. Several patients reported adherence to EB-CPG-based lifestyle advice and compliance with EB-CPG-recommended Ayurvedic antidiabetic medicine. However, some faced routine medication adherence challenges, such as occasional forgetfulness or frustration with taking multiple medicines. Patients observed notable health benefits from EB-CPG-based Ayurvedic treatment without side effects, strengthening their trust in Ayurveda as a safe approach to health. Some expressed hope for a similar approach to be applied to other health conditions. Patients noted that the monthly supply of EB-CPG-recommended Ayurvedic antidiabetic medicine was generally smooth and met their needs, though busy schedules sometimes disrupted timely pickup. They appreciated Ayurveda centers’ reminders and support, which helped maintain consistent adherence without stockpiling. Some patients expressed a desire for a written booklet to support EB-CPG-based lifestyle advice, as it would provide a helpful reference for remembering advice. As the trial ended, several patients expressed concerns about losing access to EB-CPG-recommended Ayurvedic antidiabetic medicine that had benefited them, with many hoping for continued support to sustain their health improvements.

#### Ease and difficulties of monthly follow-ups

Ayurvedic practitioners highlighted the importance of trust-building and patient prioritization in improving follow-up adherence. Regular follow-ups, timely medication, and monthly blood tests helped maintain patient engagement. However, challenges arose due to scheduling difficulties, long travel distances for some patients, and delays from local events. While regular communication could reduce dropouts, it was often impractical for practitioners to coordinate follow-ups due to their busy clinical schedules.

Patients highlighted that monthly follow-ups were manageable for those nearby and familiar with Ayurveda centers. However, others faced logistical and health-related challenges, particularly when balancing travel, health conditions, and household duties. Supportive measures from centers, like scheduling flexibility and reminders, helped but didn’t fully resolve these difficulties for everyone. Some had poor comprehension of the processes, particularly around blood tests, indicating areas for clearer communication.

#### Pros and cons of maintaining patient diaries for assessing compliance with EB-CPG-recommended Ayurvedic antidiabetic medicine

Some Ayurvedic practitioners found patient diaries helpful in tracking medication adherence. However, others noted challenges in patient compliance, especially among those with low literacy or limited understanding of the diary’s purpose. For data accuracy, some patients forgot or misreported entries, prompting practitioners to monitor pill counts and engage directly with patients to verify adherence.

Patients pointed out that keeping a diary was easy for some but challenging for others due to busy schedules or limited literacy. Forgetfulness sometimes led to delayed entries, and logistical issues, such as diary size, space, or availability, affected adherence tracking. Maintaining the diary was stressful for them.

### Themes specific to Ayurvedic practitioners

#### Ayurvedic practitioners serve diverse populations

Ayurvedic practitioners mentioned that they treat patients from diverse socioeconomic backgrounds, with a broad age range, including young adults and older individuals.

#### Patients’ motivations for choosing Ayurveda over Western medicine

Ayurvedic practitioners noted that many patients prefer Ayurvedic treatments over Western medicine options, believing they have no side effects. Positive experiences from others drive further interest. Additionally, patients on Western medicine often seek Ayurveda due to uncontrolled blood glucose, fear of insulin injections, and high treatment costs.

#### Ayurvedic practitioners’ motivations for participating in the trial

Ayurvedic practitioners mentioned that they were motivated to join the trial because they valued evidence-based clinical practice and saw the trial as beneficial both for the advancement of Ayurveda as a traditional medical system and for their own professional development. Some were drawn by their growing interest in research, particularly international studies on noncommunicable diseases, including diabetes. They felt research was needed to improve disease management using Ayurveda, particularly around treatment protocols (EB-CPGs). They also recognized the historical significance of the trial in Nepal, seeing it as an opportunity to establish solid evidence to attract and assure patients of Ayurveda’s benefits.

#### Balancing clinical duties with the trial demands

Several Ayurvedic practitioners faced challenges balancing their clinical duties with the trial demands, often viewing research as an added workload. High patient volumes required them to work longer hours or forgo personal time, though they found value in the experience. Some noted that additional staff support could have eased the burden.

#### Confusion regarding roles and responsibilities in the trial

Many Ayurvedic practitioners experienced confusion regarding trial roles and responsibilities, both among themselves and when contacting the study coordination team. This underscores the need for clearer communication about roles from the outset and a more substantial commitment to responsibilities throughout the trial.

#### Issues in understanding the trial processes and documentation

Several Ayurvedic practitioners found trial processes and documentation challenging, citing confusion around protocols and the complexity of paperwork. While initial training helped clarify some aspects, they still struggled to explain the research process to patients and felt that lengthy documentation was an additional burden. This highlights the need for regular, practice-oriented training, and reduced paperwork to improve trial efficiency.

#### Issues in the trial support and communication

Ayurvedic practitioners appreciated the support and communication from the study coordination team, but several others also noted that the support and communication were inadequate. They highlighted the need for frequent follow-ups and monitoring, especially for those with less research experience, to maintain consistent support and communication throughout the trial.

#### Issues in coordination with the trial data collectors/outcome assessors

Ayurvedic practitioners noted that while data collectors/outcome assessors were considerate of patients’ availability, coordinating timing with them proved challenging due to their preexisting schedules.

#### Trial-related financial and other resource issues

Several Ayurvedic practitioners faced financial and other resource challenges in the trial, including delayed payments, out-of-pocket expenses due to billing procedures, and delays in receiving trial-related materials.

## Discussion

This qualitative study explored the experiences and perspectives of Ayurvedic practitioners and patients in Nepal concerning two treatment groups for T2DM management—EB-CPG-based care versus usual clinical practice—and their participation in the feasibility trial. Common themes identified across both groups included facilitators and challenges in patient recruitment for the trial, challenges in providing and receiving usual clinical care, experiences of providing and receiving EB-CPG-based care (which were predominantly positive), ease and challenges of monthly follow-ups, and pros and cons of maintaining patient diaries to assess compliance with EB-CPG-recommended Ayurvedic antidiabetic medicine. Themes specific to practitioners included the diverse populations they serve; patients’ motivations for choosing Ayurveda over Western medicine; their motivations for participating in the trial; balancing clinical duties with the trial demands; confusion about trial roles and responsibilities; and other trial-related issues such as understanding processes and documentation, support and communication, coordination with data collectors, and finances and other resources.

Diverse populations served by Ayurvedic practitioners in South Asia and patients’ motivations for choosing Ayurveda over Western medicine are well documented.^10–15^ The same is true for challenges encountered in routine Ayurvedic practice.^13,15,18–29^ In this study, practitioners similarly reported inconsistencies in clinical practice, including unclear lifestyle recommendations and limited knowledge of effective and safe Ayurvedic medicines. Issues with Ayurvedic medicine quality, effectiveness, form, and availability contributed to patient dissatisfaction and poor adherence. Patients echoed these concerns, citing confusion from inconsistent clinical care, vague lifestyle advice, and unclear medication instructions, as well as challenges accessing quality and effective Ayurvedic medicines.

EB-CPGs have demonstrated the potential to enhance care quality and health outcomes, as shown in multiple systematic reviews.^45–48^ Several countries—particularly high-income nations—have successfully implemented EB-CPGs, with similar examples now emerging from low- and middle-income countries (LMICs).^49^ Ours is the first documented study to explore the use of an EB-CPG in Ayurveda in general and for the management of T2DM in particular.^50^ This qualitative study provided rich insights into the acceptability, perceived impact, challenges, and future scope of implementing an EB-CPG in routine Ayurvedic practice.

Ayurvedic practitioners valued the structured, comprehensive, and clear nature of the EB-CPG and its supporting resources, such as the training provided and the structured booklet for recording case notes. We employed a range of strategies to promote uptake and adherence to the EB-CPG, consistent with findings from an umbrella review on effective implementation strategies.^49^ This is crucial because developing and disseminating EB-CPGs alone is insufficient to achieve meaningful improvements in practice; success depends on active, context-sensitive implementation tailored to specific clinical settings.^45–49,51–54^ Practitioners emphasized that adherence to the EB-CPG minimized errors and ensured consistency in T2DM management and adherence to the care pathway. Although the primary focus of this study was not on practitioners’ soft skills, both practitioners and patients observed positive changes in this area linked to the implementation of the EB-CPG. Soft skills—such as confident communication and the ability to deliver compassionate, patient-centered care—are essential for improving health outcomes and fostering trust.^55,56^ Patients also appreciated care provided through the EB-CPG-based approach and reported perceived health benefits from adherence to practitioners’ advice based on the EB-CPG.

Our study findings on care processes and patient outcomes are crucial for assessing improvements in care quality, as emphasized in the Donabedian model.^57^ Another important dimension—the structural aspect—was largely addressed through the resources provided during the trial. Although this study was not a drug trial and we could not continue providing access to the same EB-CPG-recommended Ayurvedic antidiabetic medicine posttrial (as requested by several intervention-group patients wishing to sustain their health improvements), Ayurvedic practitioners were supported in transitioning patients to available healthcare facilities and medicines. This approach ensures ethical clinical research practices and helps maintain public trust.^58^ Several practitioners reported that the EB-CPG became an integral part of their routine clinical practice posttrial and that they continued delivering EB-CPG-based care. Some practitioners and patients suggested developing a written booklet to support EB-CPG-based lifestyle guidance, and several recommended applying a similar EB-CPG approach to manage other health conditions.

Several systematic reviews have synthesized trial conduct-related issues, including participant recruitment and retention, and suggested potential solutions to address these challenges.^59–66^ Our study findings align with those of these reviews, indicating that these are global challenges in trial conduct, particularly in LMICs like Nepal. Our previous trial-related work in India, a neighboring LMIC, also demonstrated similar facilitators and challenges.^67–69^ Although our participating Ayurvedic practitioners were motivated and interested in research and evidence-based healthcare, most of our sites (Ayurveda centers) had little to no experience with research and were new to clinical trials. Additionally, their clinical workload was high. Therefore, there is a need to provide them with continuous, high-quality trial-related training and support from the study coordination team.^70^ Simplifying trial conduct, particularly reducing trial-related workload, will improve trial efficiency—for example, by minimizing nonessential paperwork.^71,72^

In this study, Ayurvedic practitioners suggested adjusting the trial eligibility criteria in the definitive trial to better align with Ayurvedic treatment capabilities, specifically by increasing the upper glycated hemoglobin (HbA1c) limit from <9% (initially, it was <7.5%) to 10%. As a feasibility trial, we were extra cautious, setting a lower cut-off arbitrarily for patient safety, without specific scientific evidence to support it. Although our definitive trial will not test a new drug, it will be a pragmatic study addressing a real-world issue, where people with high blood glucose levels continue to prefer Ayurveda, as observed in our feasibility trial. Consequently, in the definitive trial, patients in the control group will continue to receive usual clinical care, including commonly prescribed Ayurvedic formulations, while those in the intervention group will receive EB-CPG-based care, including EB-CPG-recommended Ayurvedic antidiabetic medicine. Importantly, we will have a robust trial conduct and governance structure.

Another major challenge both Ayurvedic practitioners and patients reported was maintaining patient diaries to assess medication compliance. Given that the definitive trial will be pragmatic on disease management and will not test a new drug, in which medication compliance is assessed using patient diaries, less burdensome methods of assessing adherence could be used.^73^ These alternatives would improve the trial experience for everyone involved, such as using the Adherence to Refills and Medications Scale.^73–75^ A similar challenge was posed by monthly follow-ups. In the definitive trial, long-term follow-ups will be conducted, which are more important in this chronic condition than monthly follow-ups.^2^ That said, we will have the process in place for reporting adverse events and serious adverse events during nonscheduled visits.

To our knowledge, this is the first qualitative study to explore using an innovative approach in Ayurvedic management of T2DM, capturing the experiences and perspectives of both healthcare providers and a diverse range of patients. Additionally, trial-based qualitative evaluations remain extremely limited in Nepal. Regarding generalizability, our qualitative sample was drawn from the feasibility trial population, not the broader national population. For instance, we interviewed all Ayurvedic practitioners who participated in the trial and 25 of the 121 enrolled patients. While the study does not aim for statistical generalizability, it is analytically generalizable to the feasibility trial context. Although face-to-face interviews often generate richer data due to effective icebreaking and the ability to observe body language and facial expressions, several interviews were conducted by telephone to address accessibility issues, particularly with participants outside Kathmandu Valley. Evidence suggests that the quality of data collected via face-to-face and telephone interviews is not necessarily different.^76,77^ Although interviewers made efforts to build rapport with participants before interviews, some telephone interviews were shorter, especially with those who had declined to participate in the trial or who were recruited but later withdrew. Reaching out to these individuals proved challenging, and as a result, only a small number of them were interviewed. There are potential limitations related to recall and social desirability bias. Since interviews were conducted at the end of participants’ involvement in the trial, some individuals may not have recalled their experiences in full detail or with complete accuracy. Additionally, both patients and Ayurvedic practitioners may have provided responses they believed were expected or viewed favorably by the research team, particularly regarding the intervention or the trial overall. We sought to minimize these biases by using open-ended, nonleading questions and trained interviewers and by assuring participants that their responses would remain confidential and would not affect their care or professional roles.

In conclusion, the EB-CPG-based approach for managing T2DM by Ayurvedic practitioners in Nepal was well received and perceived as more beneficial than usual clinical practice. Trial experiences and perspectives were largely positive; however, several challenges related to trial conduct should be addressed in future studies, including the definitive cluster trial.

## Declarations

**Ethics approval and consent to participate:** Ethics approval for the study was obtained from the Research Ethics Committee, Faculty of Medicine and Health Sciences, University of Nottingham, UK (511-2003) and the Ethical Review Board, NHRC, Nepal (66/2022). The study was conducted in accordance with the Declaration of Helsinki. Participants provided consent to participate in the study. The feasibility trial was registered with a clinical trials registry (ClinicalTrials.gov Identifier: NCT05259735; first posted on March 2, 2022). It also received a clinical trial license from the Department of Drug Administration in Nepal.

**Consent for publication:** Participants provided consent to publish the study findings.

**Availability of data and materials:** The data that support the findings of this study are not openly available due to reasons of sensitivity and are available from the corresponding author upon reasonable request. Data are located in controlled access data storage at the University of Nottingham.
